# Comparison of Peritoneal Carcinomatosis Scoring Methods to Assess Resectability in Ovarian Carcinoma

**DOI:** 10.7759/cureus.100086

**Published:** 2025-12-25

**Authors:** Joju Antony Sebastian, Santhoshkumar Bandegudda, Kalla B Muralidhar

**Affiliations:** 1 Department of Surgical Oncology, Amala Institute of Medical Sciences, Thrissur, IND; 2 Department of Surgical Oncology, Prakriya Hospitals, Bengaluru, IND; 3 Department of Surgical Oncology and Laparoscopy, Mahatma Gandhi Cancer Hospital and Research Institute, Visakhapatnam, IND

**Keywords:** carcinoma ovary, cytoreductive surgery, epithelial ovarian cancer, fagotti score, peritoneal carcinomatosis index (pci), resectability assessment

## Abstract

Objective

Comparison of peritoneal carcinomatosis scoring methods in carcinoma of the ovary and evaluation of their prediction of complete cytoreduction in patients with stage III epithelial ovarian cancer.

Methods

This prospective observational study included 100 patients with stage III epithelial ovarian cancer treated at a tertiary cancer center from July 2020 to October 2021. Preoperative assessment involved USG and contrast-enhanced CT (CECT) to calculate the Peritoneal Carcinomatosis Index (PCI). Diagnostic laparoscopy was performed to record the Fagotti score. Intraoperatively, surgical PCI was determined, followed by cytoreductive surgery. Correlation between imaging-derived and surgical PCI scores was analyzed using Spearman’s correlation. Receiver operating characteristic (ROC) analysis was performed to identify PCI cutoff values predictive of complete cytoreduction.

Results

The mean surgical PCI was 8.05, while the mean CT-PCI and USG-PCI were 5.63 and 4.21, respectively. CT-PCI showed a stronger correlation with surgical PCI (ρ = 0.867) than USG-PCI (ρ = 0.750), both statistically significant (p < 0.001). The majority of patients (73%) had a Fagotti score of 0, and none scored ≥8. Complete cytoreduction was achieved in 98% of cases. A surgical PCI cutoff of 14.5 predicted complete cytoreduction with 84.7% sensitivity and 100% specificity. CT demonstrated higher accuracy than USG in detecting disease across abdominal regions, especially outside the pelvis; both were limited in identifying small bowel involvement.

Conclusions

CT-based PCI is the most reliable noninvasive modality for preoperative assessment of peritoneal disease burden. In select post-neoadjuvant patients with favorable imaging, laparoscopy may be omitted, optimizing surgical planning and outcomes.

## Introduction

Ovarian cancer is a major global health concern, ranking as the seventh leading cause of cancer-related mortality among women worldwide [1]. According to the GLOBOCAN 2020 statistics, ovarian cancer is the eighth most common cancer overall and the third most prevalent cancer among women in India [2]. Despite advancements in medical research, ovarian cancer remains challenging due to its asymptomatic nature in early stages and its tendency to be diagnosed at an advanced stage, significantly impacting survival outcomes [3].

More than 90% of malignant ovarian tumors originate from epithelial cells, while sex cord-stromal tumors and germ cell tumors account for 5-6% and 2-3% of cases, respectively [4]. Based on histopathology, immunohistochemistry, and molecular profiling, epithelial ovarian cancer is further classified into five subtypes: high-grade serous carcinoma (HGSC), which constitutes approximately 70% of cases, followed by endometrioid (10%), clear cell (10%), mucinous (3%), and low-grade serous carcinoma (LGSC) [5]. These subtypes exhibit distinct molecular characteristics and responses to treatment, emphasizing the need for personalized therapeutic approaches [6].

The standard treatment for epithelial ovarian cancer involves cytoreductive surgery and chemotherapy. Traditionally, primary cytoreduction followed by adjuvant chemotherapy was the preferred approach [7]. However, in cases of advanced disease, neoadjuvant chemotherapy (NACT) followed by interval cytoreduction surgery has emerged as an alternative strategy [8]. Several retrospective and prospective studies have shown that survival outcomes after NACT are not inferior to those following primary cytoreduction, making it a viable option for patients with extensive tumor burden [9-11].

The primary objective of cytoreductive surgery is to achieve complete cytoreduction, defined as the absence of macroscopic residual tumor at the end of surgery. Achieving complete cytoreduction has been strongly associated with improved overall survival and progression-free survival [12,13]. Incomplete cytoreduction leads to a poorer prognosis, reinforcing the importance of accurate preoperative assessment to determine the likelihood of achieving optimal cytoreduction [14].

Several tools have been developed to evaluate tumor burden and predict resectability. The Peritoneal Carcinomatosis Index (PCI), introduced by Jacquet and Sugarbaker [15,16], remains one of the most widely used systems for quantifying peritoneal dissemination. PCI divides the abdominopelvic cavity into 13 regions and assigns a score based on tumor size, with higher scores indicating extensive disease burden and lower likelihood of achieving optimal cytoreduction [17].

Imaging techniques, particularly CT and ultrasound, play a crucial role in detecting peritoneal carcinomatosis and guiding treatment decisions. Studies comparing ultrasound and CT have demonstrated similar accuracy when performed by experienced specialists [18,19]. However, due to limitations in imaging techniques, laparoscopy has been explored as an additional tool for preoperative assessment. Fagotti et al. proposed a laparoscopic scoring model that includes variables such as omental cake, peritoneal carcinomatosis, and bowel infiltration to predict suboptimal cytoreduction with high specificity [12,14].

A PCI score greater than 20 has been identified as a strong predictor of suboptimal cytoreduction, with higher scores correlating with increased surgical morbidity [17,20]. Therefore, a multidisciplinary approach integrating clinical, radiological, and laparoscopic assessments is essential to tailor treatment strategies and optimize patient outcomes in advanced ovarian cancer.

## Materials and methods

Study design and setting

This single-institution, prospective observational study was conducted at the Departments of Surgical Oncology, Radiology, and Pathology, Basavatarakam Indo-American Cancer Hospital & Research Institute, Hyderabad, India, between July 1, 2020, and October 31, 2021. The research proposal and thesis protocol of the Diplomate of National Board (DNB) candidate were first reviewed and approved by the Scientific Research Committee of the institute in its meeting held on November 12, 2019, and subsequently by the Institutional Ethics Committee (IEC) in its meeting held on June 4, 2020. Informed consent was obtained from all participants prior to enrollment.

Study population

A total of 100 patients diagnosed with epithelial ovarian cancer with peritoneal involvement were included in the study. Eligible patients were those with stage III epithelial ovarian carcinoma planned for either primary cytoreductive surgery or NACT followed by interval cytoreductive surgery. Patients with recurrent disease, stage IV disease, non-epithelial malignancies, or borderline ovarian tumors were excluded.

Preoperative evaluation

All patients underwent a detailed clinical assessment and preoperative imaging. USG was conducted one week prior to surgery using a standardized protocol by an experienced radiologist. The PCI was calculated for 13 abdominopelvic regions as per the method described by Jacquet and Sugarbaker [16]. Contrast-enhanced CT (CECT) scans of the abdomen and pelvis were performed for all patients, and PCI was independently calculated based on radiologic findings. The PCI values derived from USG and CT were recorded and later correlated with intraoperative findings.

Laparoscopic evaluation (Fagotti scoring)

Under general anesthesia, diagnostic laparoscopy was performed before definitive cytoreduction. A 1.5-2 cm supra-umbilical incision was made to establish pneumoperitoneum using an open technique. Ascitic fluid, if present, was aspirated and sent for cytological analysis. An additional 5-mm ancillary port was placed bilaterally where feasible. A systematic inspection of the peritoneal cavity was conducted, including the diaphragm, liver surface, bowel loops, omentum, mesentery, and pelvis. The Fagotti score (0-14) was recorded based on six parameters: diaphragmatic involvement, omental cake, mesenteric retraction, peritoneal carcinomatosis, stomach infiltration, and liver metastases, as described by Fagotti et al. [12].

Surgical procedure and intraoperative PCI assessment

Following laparoscopy, a midline laparotomy was performed. The surgical PCI was determined intraoperatively by evaluating disease distribution across the 13 abdominopelvic regions. Complete or optimal cytoreduction was attempted in all patients, with the surgical goal being no visible residual disease (R0 resection). The detection rates and accuracy of CT- and USG-based PCI assessments were compared with the surgical PCI findings to assess diagnostic concordance.

Statistical analysis

Data were analyzed using IBM SPSS Statistics for Windows, Version 20 (released 2011; IBM Corp., Armonk, New York, United States). Continuous variables were expressed as mean ± SD and categorical variables as frequencies and percentages. The Spearman rank correlation was used to evaluate the relationship between imaging-derived PCI (CT and USG) and surgical PCI. The Wilcoxon signed-rank test was applied for non-normally distributed continuous variables. Receiver operating characteristic (ROC) curve analysis was performed to determine PCI cut-off values predictive of complete cytoreduction, and sensitivity and specificity were calculated. A p-value < 0.05 was considered statistically significant.

## Results

The study included 100 patients with stage III epithelial ovarian cancer who underwent cytoreductive surgery between July 2020 and October 2021. The mean age of patients was 52.43 ± 11.44 years (range: 16-74 years). A majority of the patients (69%) were below 60 years of age, while 31% were 60 years or older. Most patients (64%) were postmenopausal (Table 1).

**Table 1:** Demographic characteristics of the study population

Variable	Category	Number (N), (%)
Age (years)	< 60	69 (69%)
	≥ 60	31 (31%)
Menopausal Status	Premenopausal	36 (36%)
	Postmenopausal	64 (64%)

Histopathological findings

**Figure 1:**
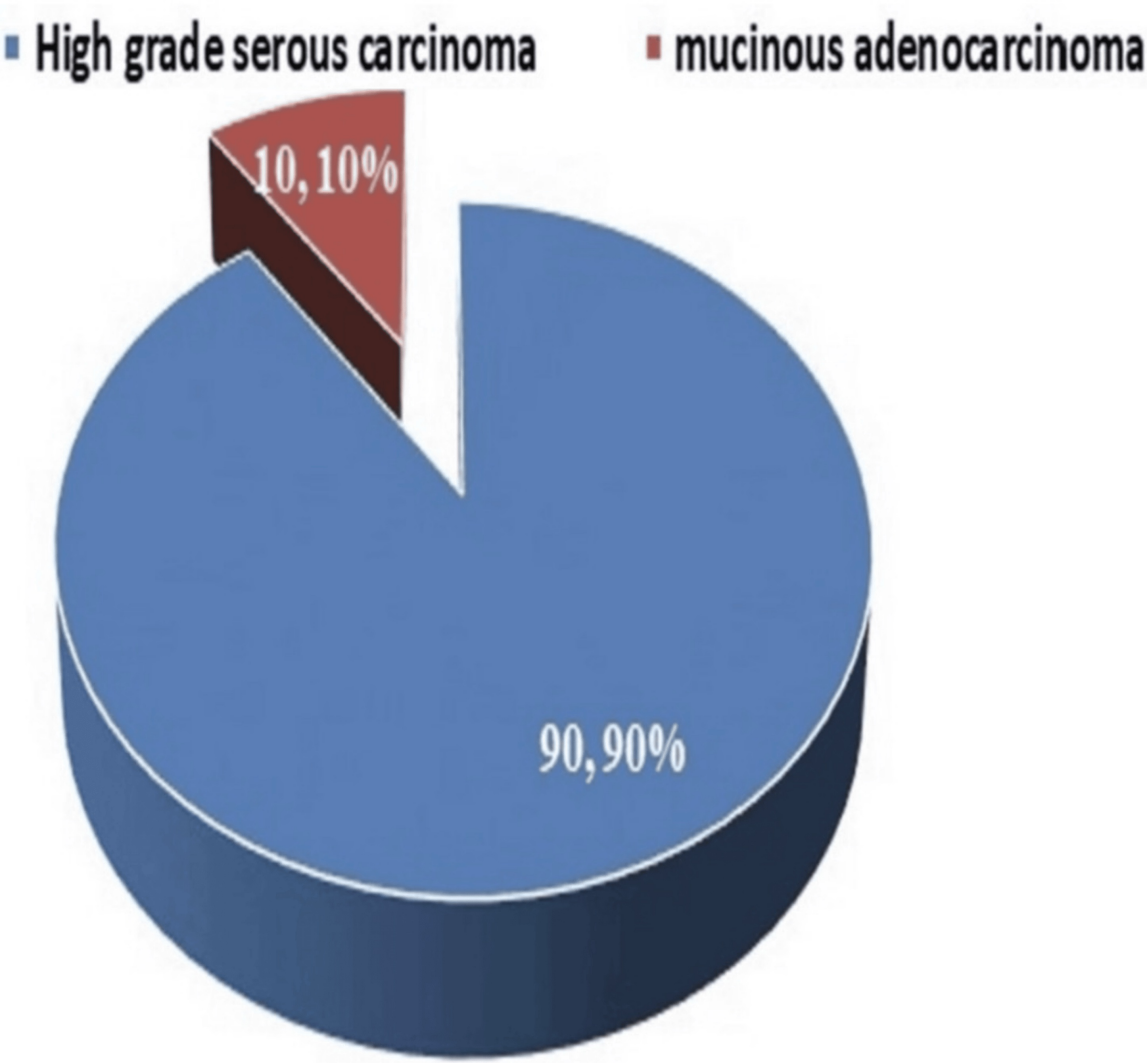
Distribution of histology of patients

The most common histological subtype identified was HGSC, accounting for 90% of cases, while the remaining 10% comprised mucinous adenocarcinoma (Figure 1).

PCI scores

The PCI was evaluated using USG, CT, and surgical assessment. The mean PCI scores from each modality are in Table 2.

**Table 2:** Mean PCI scores from different imaging modalities PCI: Peritoneal Carcinomatosis Index

PCI Scores (N = 100)	Mean ± SD	Minimum–Maximum
USG	4.21±3.086	0-16
CT	5.63±4.189	0-22
Surgical	8.05±5.591	2-24

The mean surgical PCI score was 8, while the mean USG-based PCI was 4, and the mean CT-based PCI was 6, demonstrating an increasing trend in disease burden detection accuracy from USG to CT to surgical findings.

Fagotti score

The Fagotti score was assessed in all patients. The majority of patients (73%) had a score of 0. The mean Fagotti score was 0.74 ± 1.41, with values ranging from 0 to 6. The distribution of Fagotti scores among the study population is summarized in Table 3.

**Table 3:** Distribution of Fagotti scores among study patients

Fagotti Score	Number of Patients, n (%)
0	73 (73%)
2	20 (20%)
4	4 (4%)
6	3 (3%)

Disease distribution

**Figure 2:**
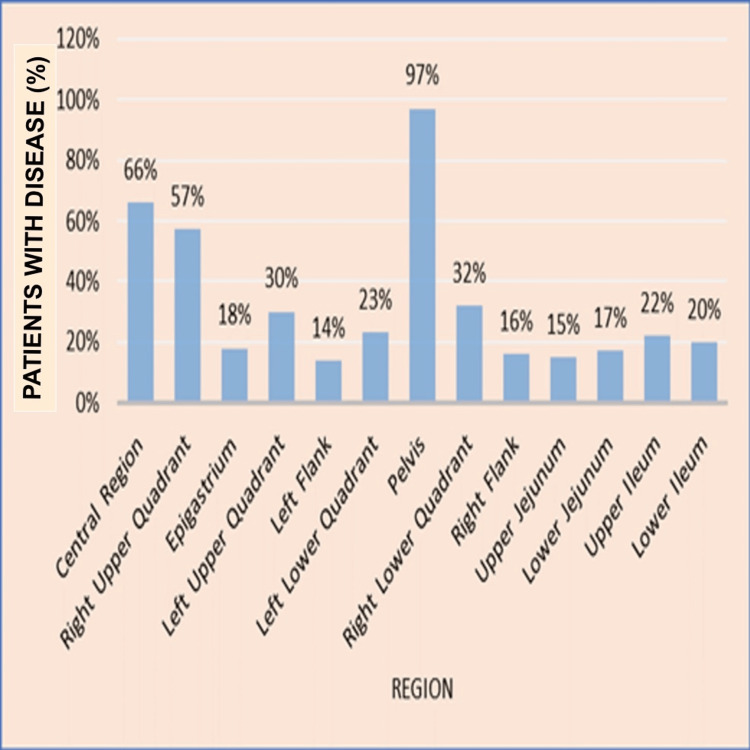
Bar graph representing disease distribution

The extent of disease involvement was analyzed for different anatomical regions. The highest disease burden was observed in the pelvic region (97%), while the epigastrium had the lowest involvement (18%) (Figure 2).

Correlation between imaging-based PCI and surgical PCI

USG PCI vs. Surgical PCI

**Figure 3:**
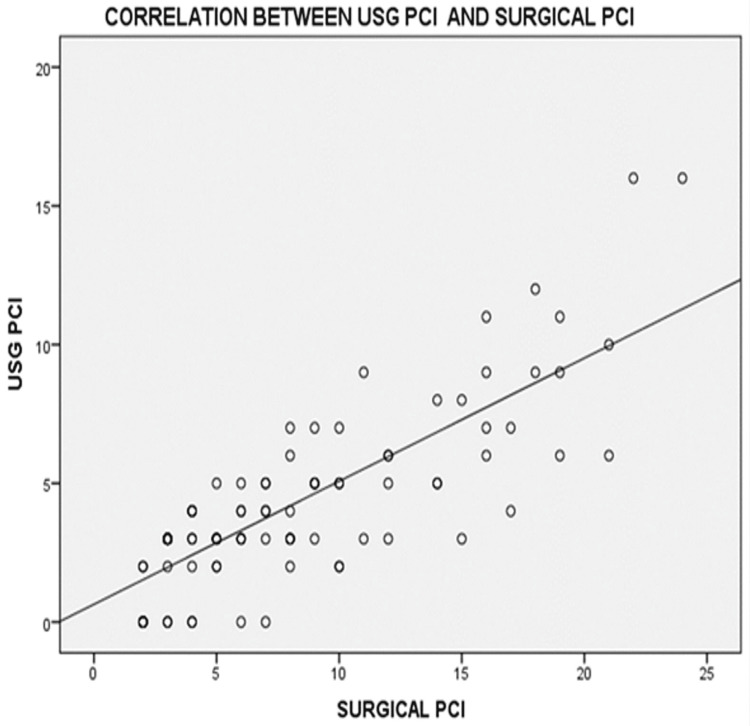
Scatter plots showing correlations between USG PCI and surgical PCI PCI: Peritoneal Carcinomatosis Index

A significant positive correlation was observed between USG PCI and surgical PCI, with a Spearman’s rho correlation coefficient of 0.750 (p < 0.001) (Figure 3).

CT-Based PCI vs. Surgical PCI

**Figure 4:**
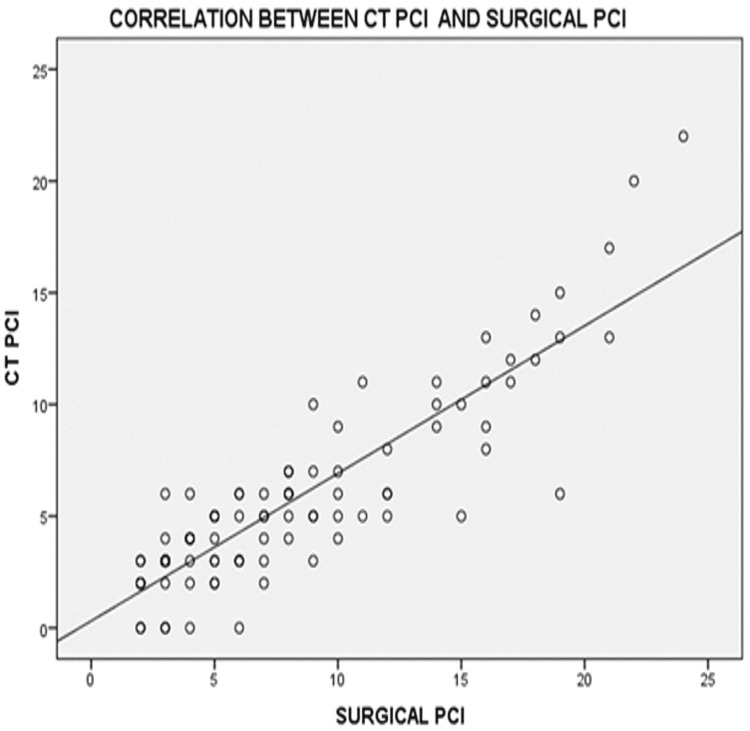
Scatter plots showing correlations between CT PCI and surgical PCI PCI: Peritoneal Carcinomatosis Index

CT-based PCI demonstrated a stronger positive correlation with surgical PCI, with a Spearman’s rho correlation coefficient of 0.867 (p < 0.001). (Figure 4)

Determination of surgical PCI cutoff and cytoreduction success

A surgical PCI cutoff value of 14.5 was determined for predicting complete cytoreduction. The area under the curve (AUC) was 0.926, with a sensitivity of 84.7% and a specificity of 100%.

**Figure 5:**
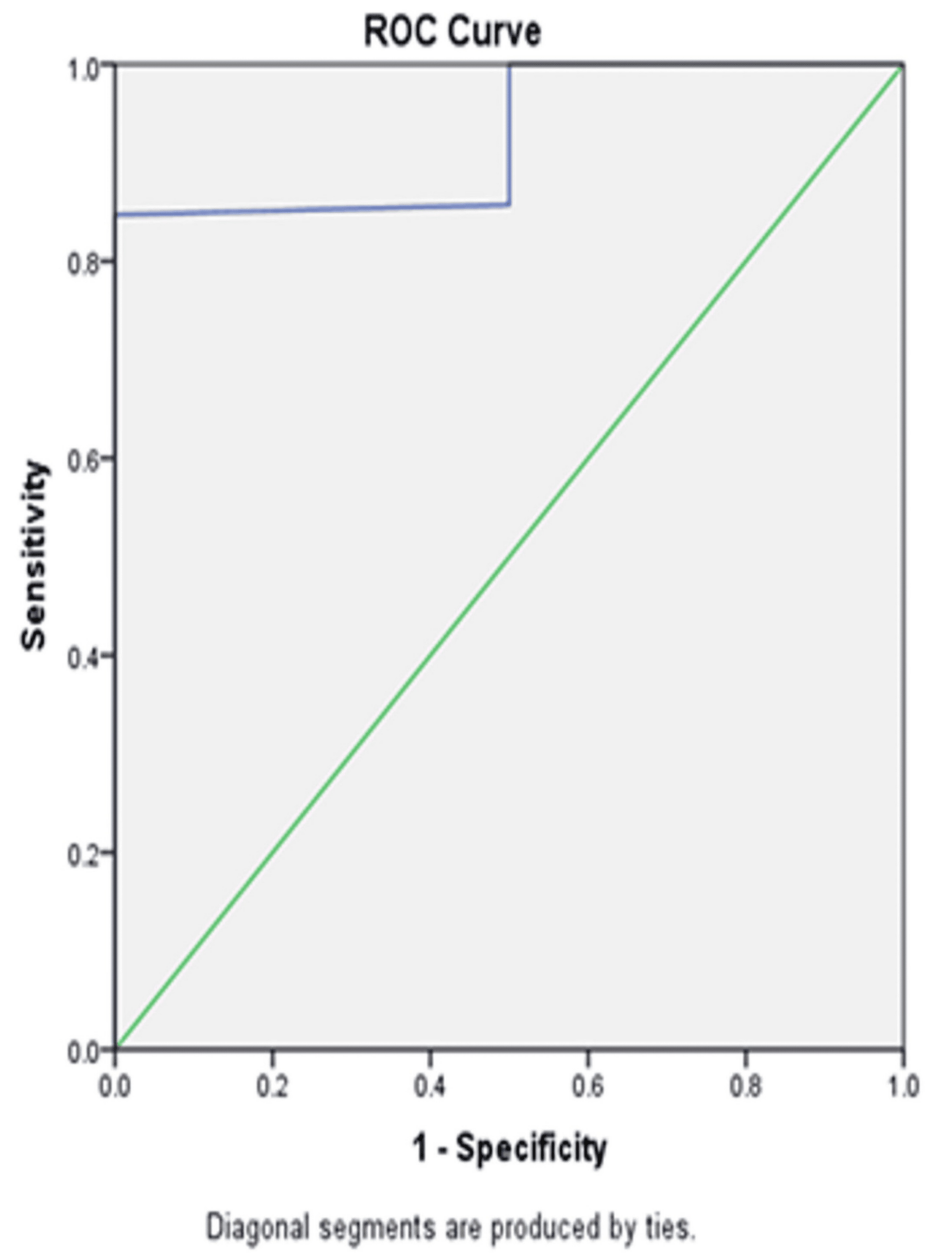
ROC curve illustrating the diagnostic accuracy of the surgical PCI cutoff for predicting complete cytoreduction PCI: Peritoneal Carcinomatosis Index; ROC: receiver operating characteristic

Among patients with surgical PCI < 14.5, 100% achieved complete cytoreduction. In contrast, 88% of patients with surgical PCI ≥ 14.5 also achieved complete cytoreduction, showing a statistically significant association (p < 0.001) (Figure 5).

Comparison of imaging modalities for disease detection

The accuracy of USG and CT scans in detecting disease across different abdominal regions was compared with surgical findings (Table 4). Overall, CECT scans showed higher accuracy than USG for disease burden detection; both USG and CECT scans showed comparable accuracy in detecting disease in the pelvic region; and a CECT scan performed slightly better than USG in detecting disease in the small bowel, but both had poor accuracy.

**Table 4:** Comparison of USG and CT scan accuracy for disease detection in different regions

Regions	USG-Sensitivity	USG-Specificity	USG-Positive Predictive Value	USG-Negative Predictive Value	CT- Sensitivity	CT- Specificity	CT-Positive Predictive Value	CT-Negative Predictive Value
Central – 0	53%	100%	100%	52.3%	74.2%	94.1%	96.1%	65.3%
Right upper - 1	35.1%	100%	100%	53.8%	56.1%	100%	100%	63.2%
Epigastrium - 2	22.2%	100%	100%	85.3%	55.6%	100%	100%	91%
Left upper -3	16.7%	100%	100%	73.7%	43.3%	100%	100%	80.5%
Left flank- 4	7.1%	100%	100%	86.9%	35.7%	100%	100%	90.5%
Left lower - 5	13%	100%	100%	79.4%	30.4%	100%	100%	82.8%
Pelvis -6	89.7%	100%	100%	25%	93.8%	100%	100%	33.3%
Right lower -7	34.4%	100%	100%	76.4%	46/9%	100%	100%	80%
Right flank - 8	37.5%	100%	100%	89.4%	37.5%	100%	100%	89.4%
Proximal jejunum – 9	0	100%	0	85%	6.7%	100%	100%	85.9%
Distal jejunum-10	0	100%	0	83%	5.9%	100%	100%	83.8%
Proximal ileum-11	0	100%	0	78%	9.1%	100%	100%	79.6%
Distal ileum - 12	5%	100%	100%	80.2%	20%	100%	100%	83.3%

## Discussion

The present study was a single-institutional prospective observational study with the primary aim to compare peritoneal carcinomatosis scoring methods in patients with ovarian carcinoma and to evaluate their prediction of complete cytoreduction in patients with stage III epithelial ovarian cancer. One hundred patients were enrolled in this study. The mean age of patients was 52 years, and 64% were postmenopausal, consistent with epidemiological data suggesting that ovarian cancer is more prevalent in older, postmenopausal women [21]. In terms of tumor histology, 90% had HGSC, while 10% had mucinous adenocarcinoma, supporting global data indicating HGSC as the most common subtype [5].

The median CA-125 level at diagnosis was 583 IU/ml. Of the 100 patients, 67 underwent NACT with paclitaxel and cisplatin followed by interval cytoreduction, while 33 had upfront primary cytoreduction.

Region-wise analysis revealed that the pelvis harbored the maximum disease burden (97%). Outside the pelvis, the central region (66%) and right upper quadrant (57%) showed high involvement, likely due to spread to the greater omentum and diaphragmatic deposits via preferential ascitic flow along the right paracolic gutter [22,23]. Disease involvement progressively decreased across the left and right flanks, upper and lower ileum, jejunum, and epigastrium.

Surgical extent included bilateral salpingo-oophorectomy in all patients, hysterectomy in 94%, and omentectomy in 100%. Pelvic peritonectomy was performed in 76%, appendectomy in 29%, right diaphragmatic peritonectomy in 58%, and left in 35%. Pelvic and para-aortic lymphadenectomies were done in 19% and 18%, respectively. Bowel resection was the most common organ resection (21%), followed by liver deposit excision (14%) and splenectomy (10%).

Mean PCI scores were 4 by ultrasound (range 0-16), 6 by CT (range 0-22), and 8 surgically (range 2-24). The lower mean PCI values may reflect tumor downstaging due to NACT in 67% of patients. A significant correlation was found between imaging-based and surgical PCI scores. CT-based PCI had a stronger correlation (Spearman’s rho = 0.867) compared to USG (rho = 0.750), supporting its superior accuracy in assessing peritoneal disease burden [24-26].

Fagotti et al. proposed a laparoscopy-based scoring system where a score ≥8 indicates unresectability [12]. In our study, none of the patients crossed this threshold; 73 had a score of 0, 20 scored 2, four scored 4, and three scored 6. Two patients with incomplete cytoreduction had Fagotti scores of 4 and 6 and surgical PCI scores of 15 and 24. In both cases, dense mesenteric involvement prevented complete resection.

All 67 patients who underwent interval cytoreduction achieved optimal debulking (100%). These patients also had low Fagotti scores, and laparoscopy did not alter surgical decision-making, suggesting that laparoscopy may be safely omitted in post-NACT patients with good CT responses, thereby reducing operative time, cost, and morbidity [27].

Region-wise imaging accuracy showed comparable results for pelvic disease between USG (89.7%) and CT (93.8%). CT outperformed USG in central (74.2% vs 53%) and diaphragmatic regions (55.6% vs 35.1%). Both modalities performed poorly for bowel involvement, with CT detecting disease in the proximal jejunum (6.7%), distal jejunum (5.9%), proximal ileum (9.1%), and distal ileum (20%), while USG detected 5% only in the distal ileum. These findings reflect limitations in detecting sub-centimeter lesions, typically picked up during laparoscopy or surgery [18,19,28]. Alcazar et al. reported higher accuracy rates (USG 71%, CT 75%), while our rates were slightly lower, likely due to extensive disease in our cohort [18].

Llueca et al. identified a PCI >20 as predictive of incomplete cytoreduction [17]. In our study, 100% of patients with PCI <15 achieved complete cytoreduction, while 88% of those with PCI ≥15 were also optimally cytoreduced. These findings reinforce the predictive validity of PCI and highlight the effectiveness of our institution’s case selection and surgical expertise.

Our study achieved a 98% overall complete cytoreduction rate. Among primary cytoreduction cases, the success rate was 93.9%, and for interval cytoreduction, it was 100%. These high rates may be attributed to strategic case selection and the proficiency of our surgical oncology team at this high-volume gynecologic cancer center.

Comparatively, Chi et al. from Memorial Sloan-Kettering Cancer Center (MSKCC) reported 50% optimal cytoreduction in primary and 76% in interval surgeries [29]. Medina-Franco et al. in Spain showed 35.5% and 64.5%, respectively [30]. Our results demonstrate that with a structured multi-modality approach and skilled surgical execution, significantly improved outcomes are achievable.

Limitations of the study

A major limitation of this study is the high proportion (67%) of patients who received NACT, likely contributing to a lower mean surgical PCI score and overall disease burden.

Additionally, incomplete cytoreduction was primarily due to localized, unresectable disease in the small bowel mesentery rather than high total PCI scores, suggesting that site-specific disease may be more critical than total burden in some cases. Finally, the low rate of incomplete cytoreduction limits the ability to draw strong conclusions about predictors of surgical failure.

## Conclusions

CT-based PCI provides the most accurate noninvasive estimation of peritoneal tumor burden and best predicts surgical resectability in stage III epithelial ovarian cancer. USG serves as an adjunct but has limited sensitivity outside the pelvis. In post-NACT patients with favorable CT findings, laparoscopy may be safely omitted without compromising outcomes. Integrating CT-PCI scoring into preoperative planning enhances surgical precision and patient prognosis.
